# Rehabilitative Effects of Virtual Reality Technology for Mild Cognitive Impairment: A Systematic Review With Meta-Analysis

**DOI:** 10.3389/fpsyg.2020.01811

**Published:** 2020-09-25

**Authors:** Jinlong Wu, Yudan Ma, Zhanbing Ren

**Affiliations:** ^1^Department of Physical Education, Shenzhen University, Shenzhen, China; ^2^Jilin Institute of Sport Science, Changchun, China

**Keywords:** mild cognitive impairment, virtual reality, cognitive function, meta-analysis, systematic review

## Abstract

**Objective:** To evaluate the impact of virtual reality (VR) technology on the cognitive functions (overall cognitive ability, executive function, short-term memory, and long-term memory) of people with mild cognitive impairment (MCI).

**Methods:** All major databases, including Web of Science, PubMed, Scopus, Proquest, WanFang, and CNKI, were searched to identify all relevant studies published in English or Chinese since October 28th, 2019. Two researchers independently conducted document retrieval, study selection, data extraction, and methodological quality evaluation.

**Result:** 15 randomized controlled trials were analyzed (*N* = 612 people with MCI), with the methodological quality evaluation score ranging from 5 to 7 points. A random effects model was selected to combine effect sizes. The result of the meta-analysis indicates that VR significantly enhanced MCI patients' overall cognitive ability (SMD = 0.869, 95% confidence interval [CI] = 0.330–1.407, *P* = 0.002, *I*^2^ = 86.822, *n* = 537) and executive function (SMD = 1.083, 95%, 95%CI = 00.134–2.031, *P* = 0.025, *I*^2^ = 93.748, *n* = 220). The meta-analysis indicated that after VR training, effects on short-term memory (SMD = 0.488, 95%CI = −0.108–1.084, *P* = 0.109, *I*^2^ = 62.354, *n* = 131) and long-term memory (SMD = 0.335, 95%CI = −1.194–0.863, *P* = 0.0.214, *I*^2^ = 58.868, *n* = 152) were not statistically significant.

**Conclusions:** The present meta-analysis verifies the potential rehabilitative effects of VR technology for mild cognitive impairment.

## Introduction

Mild cognitive impairment (MCI) is a syndrome. Those with MCI will have a cognitive ability level lower than would be expected for their age and education level, but it does not significantly interfere with daily living activities (Gauthier et al., 2006; Austrom and Lu, 2009; Oh and Lee, 2016). The International Working Group on mild cognitive impairment has formulated specific criteria, including: the individual is neither normal nor demented; there is evidence of cognitive deterioration, shown by either objectively measured decline over time or subjective report of decline by the self or an informant in conjunction with objective cognitive deficits; and activities of daily life are preserved and complex instrumental functions are either intact or minimally impaired (Winblad et al., 2004). Given the unclear MCI etiology and different parts of the brain being affected, patients may suffer from a range of clinical symptoms, including memory and executive function impairment, weakened language function, and decline in visuospatial skills (Albert et al., 2011). Activities related to executive functions, like multitasking and planning, can cover a range of actions (Gothe et al., 2014). Executive dysfunction results in the limitation of general activities in daily life (Marshall et al., 2011). Thus, MCI patients who are not able to execute instrumental activities may encounter early-stage loss of social participation and independence, consequently affecting their quality of life (Choi and Lee, 2019). Indeed, MCI is a significant risk factor for Alzheimer disease (AD). Among the elderly with average cognitive ability, the incidence rate of AD is about 1% annually, while that among MCI patients is as high as 14% (Hwang and Lee, 2017). Studies also report that the prevalence of MCI and AD will continue to increase (Realdon et al., 2016). As AD is irreversible and faces significant treatment difficulties, the key to preventing and treating AD is to take early prevention and intervention measures. As the most active part of AD, the MCI stage provides a “window of opportunity” for AD's prevention and treatment (Austrom and Lu, 2009). With previous studies having verified cognitive plasticity (Simon et al., 2012), to improve an individual's cognitive functions and to reduce the symptoms in the MCI stage will likely reduce the incidence rate of AD and thus lower healthcare costs and improve the individual's living quality (Jak, 2012). Therefore, it would be of importance to carry out research into the elderly with MCI and the influencing factors on their health.

Virtual reality (VR) technology, as an emerging intervention, has gradually become an adjunctive therapy of various diseases (such as cerebral palsy, depression, and Parkinson's disease) (Roosink et al., 2016). VR technology is a technology that uses the human senses (sight, touch, movement) to control a virtually-created environment. Its advantage is to stimulate the real-life experience (Rizzo et al., 2000) and provide short-term feedback (Liao Y. et al., 2019) according to the individual' s performance by creating a virtual environment. At present, AR technology can be divided into full-immersion, semi-immersion, and non-immersion; the main difference is the ratio of the immersive virtual environment to the real environment (García-Betances et al., 2015; Kim et al., 2017). In addition, VR technology allows patients to do exercise in a limited space, and thus reduces healthcare costs since it does not require the presence of therapists as much as more traditional therapies. Compared with traditional treatment, it enhances personal motivation and engagement (Kim et al., 2017; Penke et al., 2010). Moreover, VR technology does not only accurately control the environment but also adjusts the difficulty level according to the skill level of patients. Because of its accessibility and safety, it is convenient for patients to use at home for a short period and is beneficial to the implementation of VR rehabilitation (Grealy et al., 1999; Amjad et al., 2018; Hwang and Park, 2018).

Since VR could also be adjusted according to patients' needs and characteristics when they perform activities, tasks, and tests (Oliver and Phane, 2014), under a VR condition patients can feel a variety of sensory stimulations in a cozy, safe, and immersive environment, which can improve functional learning and transfer learned functions (Sanchez et al., 2013). A previous study suggested that VR technology improves cognitive (i.e., executive function, memory) and routine functions by stimulating patients' brain improvement among MCI patients. During the rehabilitation process, MCI patients are able to perform basic activities of daily living (i.e., walking and feeding) and then instrumental activities of daily living (i.e., managing finances and shopping) in virtual reality environments with the assistance of VR technology (García-Betances et al., 2015; Kim et al., 2019). In addition, the recovery of ability to perform daily living activities and improve cognition reduces the risk of Alzheimer's disease in the MCI population (Liao Y. et al., 2019).

Existing reviews on the effect of VR technology on MCI or AD patients have shown promising results for the use of VR. Studies reported that VR has small or medium effects on MCI or AD, and semi-immersion VR technology may have a better intervention effect on MCI or AD patients (Kim et al., 2019). Another review also found that computerized cognitive training or VR technology can improve the cognition, executive functions, and attention of MCI or AD patients to some extent (Coyle et al., 2015). Such actions have been discussed in a recent review summarizing the intervention effects of computer software, tablets, game consoles, and VR on MCI patients, which found that they exhibited a similar effect on the cognitive functions of MCI patients. It should be noted, however, that the differences among research designs weakens the evidence for its effectiveness. At present, the number of RCTs related to VR and MCI continues to increase. It is worth noting that Kim's study (Kim et al., 2019) included fewer randomized controlled experiments. The cognitive function only reported the overall cognitive and executive functions. Cognitive decline is a core symptom of mild cognitive dysfunction, and detailed reports are necessary. Given this, to increase the evidence that VR is useful in the rehabilitation of cognitive function in MCI patients, we conducted a systematic review in which quantitative data of cognitive outcomes (overall cognitive ability, executive functions, memory) were meta-analyzed. Utilizing meta-analyses provides an essential theoretical basis for the intervention effect of VR technology on improving the MCI cognitive function development. It provides a scientific and rational medical decision-making basis for clinicians.

## Methods

This study was carried out in accordance with PRISMA-P(Preferred Reporting Items for Systematic Reviews and Meta-Analyses Protocols) guidelines to ensure transparency of the study procedures (Shamseer et al., 2015).

### Literature Search

For the present study, we searched six databases, including Chinese specific databases WanFang and CNKI and others, including Web of Science, PubMed, Scopus, and Proquest, from inception to October 28th, 2019 for all eligible studies. The languages were limited to English and Chinese. Two researchers used two groups of keywords to conduct document retrieval: (1) In English: mild cognitive impairment, early-stage dementia, memory disorder, cognitive decline, or memory impairment; in Chinese: 轻度认知障碍(MCI), 早期痴呆(early-stage dementia), 记忆障(memory impairment), or 认知衰退(cognitive decline). (2) In English: virtual reality, virtual, augmented reality, active video games, AVG, or VR; in Chinese: 体感游(motion sensing games), 虚拟(virtual), 虚拟现实(virtual reality), or VR. The keywords were combined using AND, and were searched in “subject” or “title+abstract.” To include as much relevant research as possible, additional studies were identified by a manual search of the references of the original studies.

### Eligibility Criteria

First, the two researchers independently screened titles and abstracts and selected the studies concerning the effects of VR technology on MCI patients. Repetitive or non-relevant studies and reviews were screened out, and the full-text studies were confirmed. Next, full-text retrieval was conducted based on the eligibility criteria, including: (1) Type of study: all randomized controlled trials comparing the effects of VR technology on the cognitive functions of MCI patients; all studies are peer-reviewed publications, excluding conference abstracts and case experiments, no matter whether allocation concealment or blinding is applied. (2) Research subjects: all the research subjects were diagnosed by experienced therapists as people with MCI (all subjects are assessed using diagnostic tools such as the Montreal Cognitive Assessment score <26 (MoCAA <26), the Petersen's Criteria for Amnestic-Mild Cognitive Impairment, the Clinical Dementia Rating, and/or the Mini-Mental State Examination score greater than or equal 24 (MMSE≥24). (3) Interventions: VR technology-related designs and VR technology are the main intervention (such as VR technology compared with traditional rehabilitative treatment, VR technology + traditional rehabilitative treatment, compared with traditional rehabilitative treatment). (4) Research data: sample size, mean value, and standard deviation or *P*-value, F value, and sample size are provided. (5) Outcome indicator: indicators related to cognitive ability (such as global cognitive function, executive function, and memory). Studies that did not meet the criteria were excluded. Disagreements between the two researchers was resolved through discussion with a third reviewer.

### Descriptive Data Extraction of Eligible Studies

Descriptive data were extracted independently by the two reviewers after reading full texts. The extraction covers five aspects (reference, participant characteristics, intervention protocol, measurement, and follow-up time). For the reference of selected studies, the name of authors, year of publication, country, and language were extracted. Extracted participant characteristics included diagnostic criteria (qualification of the assessor), sample size, sex ratio, mean age (y), and educational level. If the experiment did not use intention-to-treat analysis, the number of participants is the same as the number of participants being analyzed at the end. Detailed information on the intervention protocol extracted included weekly dosage (frequency and session length) of VR, intervention duration, intervention location, VR platforms, and VR task. Cognitive function indicators include global cognitive function, executive function, short-term memory, and long-term memory. Follow-up time was extracted from the selected studies as well. The mean (M), standard deviation (SD), *F*-value, and *P*-value of the experiment group and control group at baseline and after the intervention in each eligible RCT were extracted.

### Study Quality Assessment

In this study, we used the Physiotherapy Evidence Database (PEDro) scale, which is widely used to assess the methodological quality of clinical trials in the field of physical therapy and rehabilitation (Albanese et al., 2020). The two reviewers independently used the PEDro quality assessment scale to conduct treatment evaluation of the included studies. In all selected papers, the experiment group underwent VR technology intervention, making blinding of both participants and instructors impossible. Therefore, we excluded these two items on the PEDro scale and kept nine items. In that case, the criteria on the revised version of the PEDro scale include randomization, concealed allocation, baseline equivalence, blinding of assessors, a retention rate of 85%, missing data management (intent-to-treat analysis), intergroup analysis, and point measure and measures of variability. One point was awarded if the information was explicitly presented, with a maximum of 9 points per study. One point was awarded if the information was explicitly presented, with a maximum of 9 points per study.

### Quantitative Data Extraction and Data Synthesis

To accurately calculate the combining effect size estimates of VR technology on the MCI cognitive functions, one researcher first conducted quantitative data extraction, and the other performed data verification in order to guarantee data accuracy. The data extracted include sample size as well as the M and SD of the experiment group and control group before and after the intervention. If the SD before and after the intervention was not reported, the *F*-value and *P*-value were extracted for analysis. Comprehensive Meta-Analysis V2 was used to combine effect size after data extraction, a random effect model was applied in the calculation, and the standardized mean difference (SMD) was selected as the effect-size index. The effect size indicates the degree of VR technology's influence on the MCI group's cognitive functions. The heterogeneity of selected studies was measured using *I*^2^. The larger the *I*^2^ measure, the higher the heterogeneity. The low, medium, and high levels of heterogeneity are indicated by 25, 50, and 75% *I*^2^ measure. In addition, the funnel plot of each cognitive result and Eager test were processed to evaluate publication bias, and the leave-one-out sensitivity analysis was performed to check the influence of individual study on the pooled results (Wang et al., 2009). For studies that did not provide the above-mentioned statistics, the author of the original study was contacted in an attempt to retrieve data, in order to improve the quality of this analysis.

## Results

### Studies Reach

**Figure 1:**
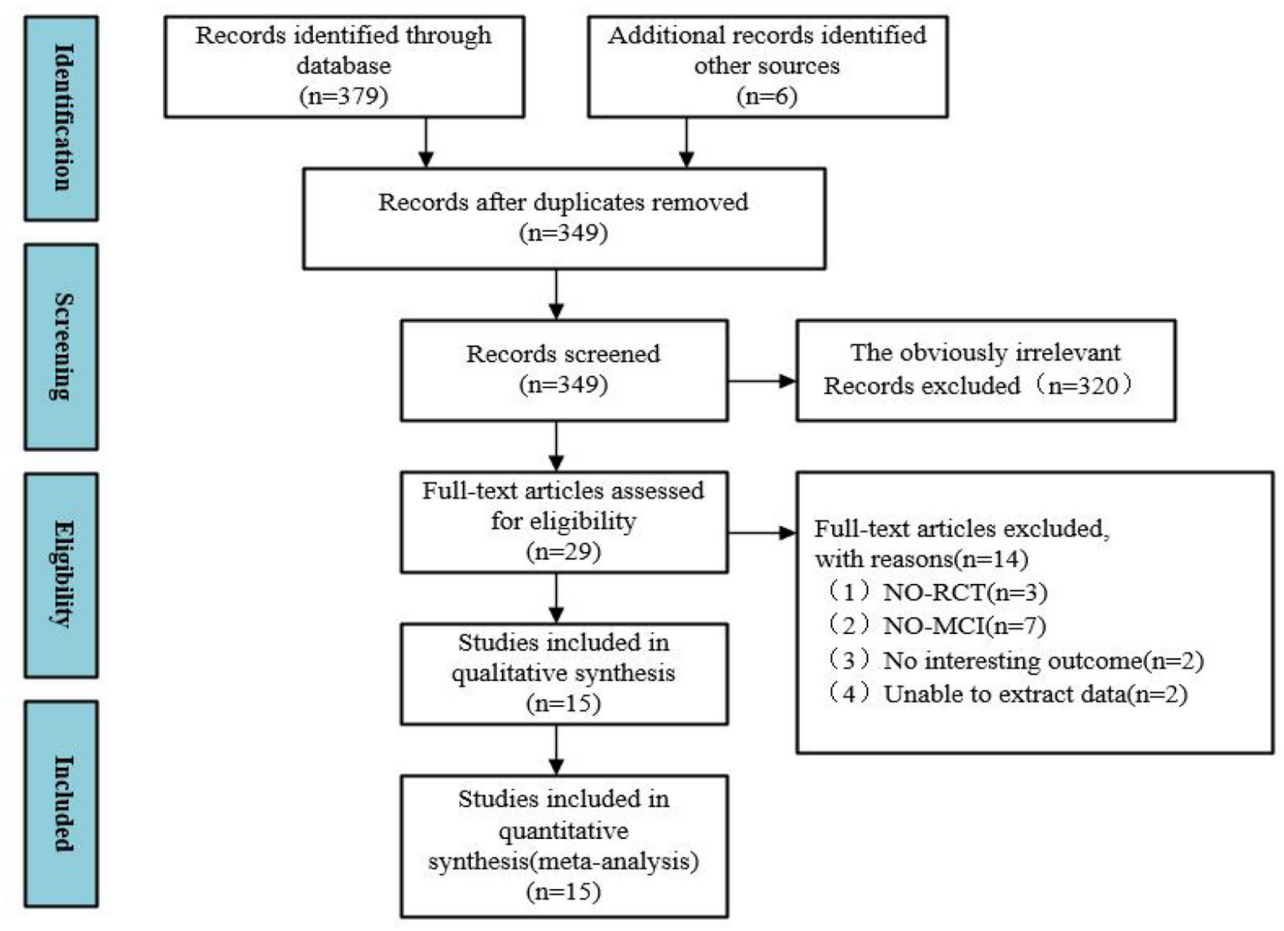
The flow of literature search and study selection.

Through databases and manual searches, 385 records were retrieved. First, repeats check was conducted in the records, and 36 repetitive texts were removed. Based on the research features, the remaining 349 texts were further screened, and 320 texts were removed as they did not meet the research criteria in this study. Next, full-text qualification assessment was performed on 29 texts, among which 14 didn't meet the eligibility criteria. Finally, 15 RCT texts were confirmed for meta-analysis. The flow of the literature search and data selection is presented in Figure 1.

### Study Characteristics

This study systematically summarizes previous studies on VR technology's effect on MCI. A total of 15 studies were included, which were published between 2010 and 2019. This suggests that, as a new intervention method, research on the cognitive benefits of VR technology to MCI patients is still in its infancy. Of the 15 studies, two studies were conducted in China (Wang and Lv, 2016; Hu et al., 2018), two in Taiwan (Liao Y. et al., 2019; Liao Y. Y. et al., 2019; Yang et al., 2019), two in the United States (Hughes et al., 2014; Schwenk et al., 2016), four in South Korea (Hwang and Lee, 2017; Hwang and Park, 2018; Choi and Lee, 2019; Park et al., 2019), and one study each in Italy (Optale et al., 2010), Belgium (Delbroek et al., 2017), Hongkong (Man et al., 2012), and Pakistan (Amjad et al., 2018). A total of 612 MCI patients were included in our analysis (age range, 59.61–87.5; one study Amjad et al., 2018 did not report the age). Most of the subjects were assessed using diagnostic tools such as MMSE and MoCA or were diagnosed as MCI by a medical professional in a hospital setting. Only one study did not report how participants were diagnosed (Amjad et al., 2018). Furthermore, the studies varied based on the education level of the participants as interventions. Seven studies (Optale et al., 2010; Man et al., 2012; Amjad et al., 2018; Hwang and Park, 2018; Choi and Lee, 2019; Park et al., 2019; Yang et al., 2019) were comprised comparisons between VR technology and conventional rehabilitation methods (including health education, memory training, computerized cognitive training, physical exercises, etc.). Another seven studies (Optale et al., 2010; Schwenk et al., 2016; Wang and Lv, 2016; Hu et al., 2018; Liao Y. et al., 2019; Liao Y. Y. et al., 2019) were comparisons between VR technology and conventional rehabilitation methods. One study (Delbroek et al., 2017) was a comparison between patients who used VR technology and patients who did not participate in rehabilitation. The duration of the intervention was between 4 and 24 weeks and the session duration was between 18 and 60 min. A summary of the included studies' characteristics is presented in Table 1.

**Table 1:** Characteristics of eligible studies.

**References**	**Country; Language**		**Participant characteristics**			**Intervention protocol**			**Measurement**		**Follow-up time**
		**Diagnostic criteria (assessor)**	**Participants (M/W)**	**Mean age(y)**	**Education level**	**Frequency**	**Duration (wk)**	**VR platforms**	**VR task**		
Optale et al. (2010)	Italy; English	VSR	EG:15(5/10) CG:16(5/11)	EG:78.5 ± 10.9 CG:81.6 ± 5	EG:5.3 ± 2.4 CG:6 ± 3.5	EG: Initial training phase, 15 min each time, 3 times every two weeks, booster training phase,15 min each time, 1 times per week (VR‵ Rauditory) CG: Music therapy	25	Virtools platform with a VR development kit	NR	Global cognitive function: MMSE,MSNExecutive function: PVF,DTP,CETShort-term memory: DSLong-term memory: VSR	6 months
Man et al. (2012)	Hong Kong; English	CDR;CMMSE	EG:20(3/17) CG:14(2/22)	EG:80.30 ±1.21 CG:80.28 ±1.31	EG: <1 year(16);1–2 years(2) >2years(2) CG: <1 year(14);1–2 years(4); >2 years(6)	EG: 30 min each time, 2–3 times per week (VR) CG: Memory training	3–5	VR environment and simple computer operations	Home setting‵ Convenience shop	Global cognitive function: MMQ-SLong-term memory: FOME-DR	No
Hughes et al. (2014)	United States, English	MMSE≥24	EG:10(2/8) CG:10(4/6)	EG:78.5 ± 7.1 CG:76.2 ± 4.3	NR	EG: 30 min per week (VR) CG: Health Education in Older Adults	24	Nintendo Wii™	Bowling, Golf, Tennis,Baseball	Global cognitive function: CAMCIExecutive function: CAMCI-B	1 year
Wang and Lv (2016)	China; Chinese	DSM	M:38 W:22	59.61 ± 8.73	NR	EG: Virtual reality training‵ Manipulating method CG: Manipulating method	NR	3D Time Difference Ranging Motion Capture	NR	Global cognitive function: MMSE,MoCA	No
Schwenk et al. (2016)	United States; English	MoCA <26	EG:12(5/7) CG:10(5/5)	EG:77.8 ± 6.9 CG:79.0 ± 10.4	EG:14.2 ± 2.3 CG:15.9 ± 2.7	EG: 45 min each time, 4 times per week (VR‵ Cognitive task) CG: Routine rehabilitation	4	A 24-inch computer screen, an interactive virtual user interface, and five inertial sensors (LegSys, BioSensics LLC; Cambridge, Massachusetts) including a triaxial accelerometer, gyroscope, and magnetomete; visuomotor rotation task‵ Virtual Obstacle-Crossing Task	NR	Global cognitive function: MoCA,Trail-A,Trail-B	No
Delbroek et al. (2017)	Belgium, English	MoCA <26	EG:10(2/8) CG:10(5/5)	EG:86.9 ± 5.6 CG:87.5 ± 6.6	NR	EG: 18 min in week 1 gradually increased to 30 min in week 5, twice a week (VR‵ Routine care) CG: No other treatment	6	BioRescue (RM Ingenierie, France)	NR	Global cognitive function: MoCA	No
Hwang and Lee (2017)	Korea; English	MMSE≥24	EG:12(4/8) CG:12(3/9)	EG:74.1 ± 6.0 CG:70.1 ± 5.3	NR	EG: 30 min each time, a total of 20 time s(VR) CG: Routine rehabilitation	4	NR	NR	Global cognitive function: VST	No
Hwang and Park (2018)	Korea; Korean	CDR;MMSE≥24	EG:20(8/12) CG:20(9/11)	EG:74.45 ± 6.20 CG:73.15 ± 5.50	U/E/J/S/C; EG:12/0/7/1/0 CG:9/11/0/0/0	EG: 30 min each time, 5 times per wee k(VR‵ Cognitive task) CG: Routine rehabilitation	6	Kinect's XBOX360 system (Microsoft Corporation, Seoul, Korea)	Beach volley ball,Ping-pong,Bowling,Boxing	Global cognitive function: DST	No
Hu et al. (2018)	China; Chinese	MoCA <26	EG:30(24/6) CG:30(23/7)	EG:73.6 ± 6.3 CG:74.6 ± 5.3	NR	EG: 30 minute health education +VR treatment (the initial treatment time is 5 min /time, and then gradually increased to 15 min / times), 5 times per CG: Health education‵ Routine rehabilitation	12	BioMaster virtual scene interactive rehabilitation systemcontent	VR tasks-·Housework‵ Kitchen cooking‵ Cycling simulation	Global cognitive function: MoCA	No
Amjad et al. (2018)	Pakistan; English	Hospital evalution	EG:22(2/8) CG:22(2/9)	NR	NR	EG: 5 min warm-up, 25–30 min VR treatment, and 10 min cooldown each time, 2 times per week (VR) CG: Routine rehabilitation	6	Xbox 360 Kinect (Microsoft Corporation, Redmond, WA, USA)	NR	Global cognitive function: MMSE,MoCA Executive function :TMT-A,TMT-B	No
Choi and Lee (2019)	Korea;English	MoCA <26	EG:30(5/25) CG:30(4/26)	EG:77.27± 4.37 CG:75.37 ± 3.97	NR	EG: 10 min warm-up, 40 min VR treatment, and 10 min cooldown each time, 2 times per week CG:Home exercise	6	Soft balance foam (TheraBand Exercise Station, Hadamar, Germany)‵ video editing software (Vegas Pro version 13; Sony, Tokyo, Japan)‵ screen (Model BX327; LG, Seoul, South Korea)	Virtual Kayak Paddling Exercise	Global cognitive function: MoCA,GPCOG	No
Liao Y. Y. et al. (2019)	Taiwan: English	MMSE≥24:MoCA <26	EG:18(11/7) CG:16(12/4)	EG:75.5 ± 5.2 CG:73.1 ± 6.8	EG:9.3 ± 3.8 CG:9.9 ± 2.1	EG: 40 min VR treatment and 20 min Physical cognitive therapy each time, 3 times per week CG: Physical and cognitive therapy	12	Kinect system (Microsoft Corporation, Redmond, WA, USA)	the taking mass rapid transit‵ the kitchen chef game‵ the s convenience store clerk	Global cognitive function: MoCA Executive function: EXIT-25 Memory: CVVLT	No
Liao Y. et al. (2019)	Taiwan: English	MoCA <26	EG:18(11/7) CG:16(12/4)	EG:75.5 ± 5.2 CG:73.1 ± 6.8	EG:9.3 ± 3.8 CG:9.9 ± 2.1	EG: 40 min VR treatment and 20 min Physical cognitive therapy each time, 3 times per week CG: Physical and cognitive therapy	12	Kinect system (Microsoft Corporation, Redmond, WA, USA) content includes	Taking mass rapid transit‵ Kitchen chef game convenience store clerk	Global cognitive function: TMT,SCWT	No
Yang et al. (2019)	Taiwan; English	MoCA <26	EG:33(8/25) CG:33(6/27)	EG:75.4 ± 6.6 CG:81.7 ± 7.2	U/E/J/S/C; EG:0/7/3/9/14 CG:0/10/5/9/9	EG: 45 min Virtual interactive working memory treatment esch time,3 times per week CG: Passive information therapy	12	CogniPlus software (Schuhfried GmbH, Vienna, Austria)	NR	Global cognitive function: MMSE,MoCA Short-term memory: WMS-III Long-term memory: WMS-III	3 months
Park et al. (2019)	Korea; English	MMSE-K≥24;CDR-K	EG:10(2/8) CG:11(2/9)	EG:70.5 ± 4.2 CG:72.6 ± 5.3	EG:7.09 ± 3.36 CG:7.09 ± 3.36	EG: 30 min each time,3 times per week (VR) CG: Computer-aided cognitive training	6	Mixed Reality System for Health (Kyung-Pook National University,Korea)		Executive function: TMT-B Long-term memory: WLR	no

*EG, Experimental group; CG, Control group; M, Men; W, Women; NR, Not reported; DSM, The diagnostic and statistical manual of mental disorders; MMSE, The Mini-Mental State Examination; MoCA, Montreal Cognitive Assessment; EXIT-25, Executive Interview 25; CVVLT, Chinese of the Verbbal Learning Test; TMT, Trail Making Test; SCWT, Stroop Color and Word Test; GPCOG, General Practitioner Assessment of Cognition; CDR, Clinical Dementia Rating; MMSE-k, The Korean version of Mini-Mental State Examination Korean; U/E/J/S/C, Uneducated/Elementary school/ Junior high school/Senior high school/College; DST, Digital Span Test; PVF, The Pho nemic Verbal Fluency Test; DTP, The Dual Task Performance Test; CET, The Cognitive Estimation Test; DS, The Digit Span Test; VSR, The Verbal Story Recall Test; WMS-III, The Wechsler Memory Scale—Third Edition; VST, Visual Span Tes; CDR, The Clinical Dementia Scale; MMQ-S, Multifactorial Memory Questionnaire-strategy; FOME-DR, Fuld Object Memory Evaluation; MSN, The Mental Status in Neurology; WLR, Word List Recognition; TMT-B, Trail Making Test Part B; CDR-K, The Korean version of the Clinical Dementia Rating Scale; CAMCI, The Computerized Assessment of Mild Cognitive Impairment*.

### Methodological Quality of Selected Studies

The quality of all included studies (*n* = 15) was assessed using the PEDro scale. Their score ranges from 5 to 7 points, with an average of 6.27 points. The subjects of one study (Amjad et al., 2018) were assessed at a hospital and didn't undergo an eligibility assessment. 12 studies (Man et al., 2012; Hughes et al., 2014; Schwenk et al., 2016; Wang and Lv, 2016; Delbroek et al., 2017; Hwang and Lee, 2017; Amjad et al., 2018; Hu et al., 2018; Hwang and Park, 2018; Choi and Lee, 2019; Park et al., 2019; Yang et al., 2019) didn't apply allocation concealment, 14 (Optale et al., 2010; Man et al., 2012; Hughes et al., 2014; Schwenk et al., 2016; Wang and Lv, 2016; Delbroek et al., 2017; Amjad et al., 2018; Hu et al., 2018; Hwang and Park, 2018; Choi and Lee, 2019; Liao Y. et al., 2019; Liao Y. Y. et al., 2019; Park et al., 2019; Yang et al., 2019) didn't blind the evaluator, and 14 (Optale et al., 2010; Man et al., 2012; Hughes et al., 2014; Schwenk et al., 2016; Wang and Lv, 2016; Delbroek et al., 2017; Hwang and Lee, 2017; Amjad et al., 2018; Hu et al., 2018; Hwang and Park, 2018; Choi and Lee, 2019; Liao Y. et al., 2019; Liao Y. Y. et al., 2019; Park et al., 2019) didn't perform intention-to-treat analysis (see Table 2).

**Table 2:** Quality assessment of eligible studies.

**References**	**Item 1**	**Item 2**	**Item 3**	**Item 4**	**Item 5**	**Item 6**	**Item 7**	**Item 8**	**Item 9**	**Sum score**
Optale et al. (2010)	1	1	1	1	0	1	0	1	1	7
Man et al. (2012)	1	1	0	1	0	1	0	1	1	6
Hughes et al. (2014)	1	1	0	1	0	1	0	1	1	6
Wang and Lv (2016)	1	1	0	1	0	1	0	1	1	6
Schwenk et al. (2016)	1	1	0	1	0	1	0	1	1	6
Delbroek et al. (2017)	1	1	0	1	0	1	0	1	1	6
Hwang and Lee (2017)	1	1	0	1	1	1	0	1	1	7
Hwang and Park (2018)	1	1	0	1	0	1	0	1	1	6
Hu et al. (2018)	1	1	0	1	0	1	0	1	1	6
Choi and Lee (2019)	0	1	0	1	0	1	0	1	1	5
Amjad et al. (2018)	1	1	0	1	0	1	0	1	1	6
Liao Y. Y. et al. (2019)	1	1	1	1	0	1	0	1	1	7
Liao Y. et al. (2019)	1	1	1	1	0	1	0	1	1	7
Yang et al. (2019)	1	1	0	1	0	1	1	1	1	7
Park et al. (2019)	1	1	0	1	0	1	0	1	1	6

*Indicate notes as following: Item 1, eligibility criteria; Item 2, randomization; Item 3, concealed allocation; Item 4, similar baseline; Item 5, blinding of assessors; Item 6, more than 85% retention; Item 7, missing data management (intent-to-treat analysis); Item 8, between-group comparison; Item 9, point measure and measures of variability; 1, explicitly described and present in details; 0, absent, inadequately described, or unclear*.

### Effects of VR on Global Cognitive Function

**Figure 2:**
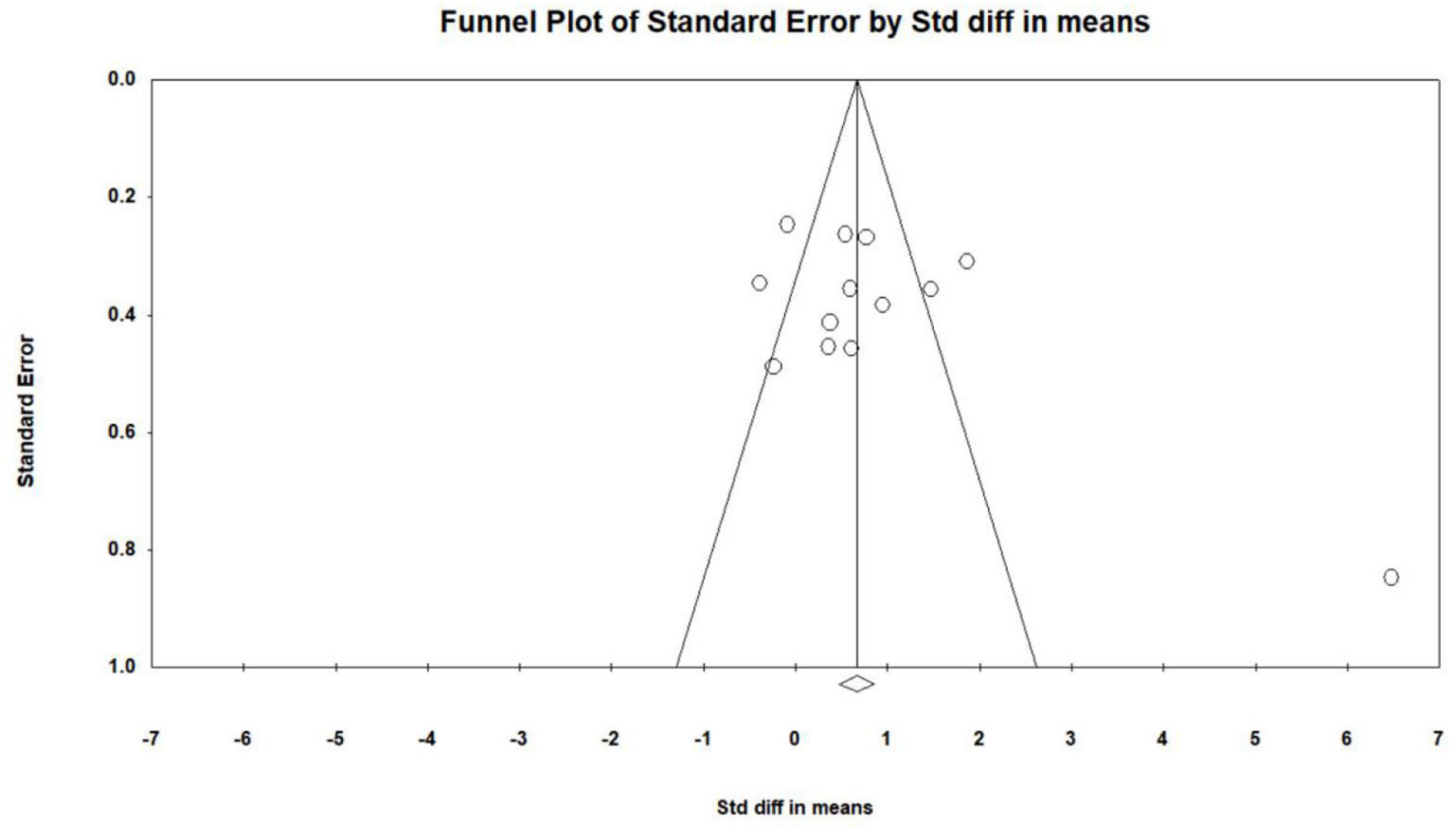
The publication bias of overall cognition (funnel plot).

**Figure 3:**
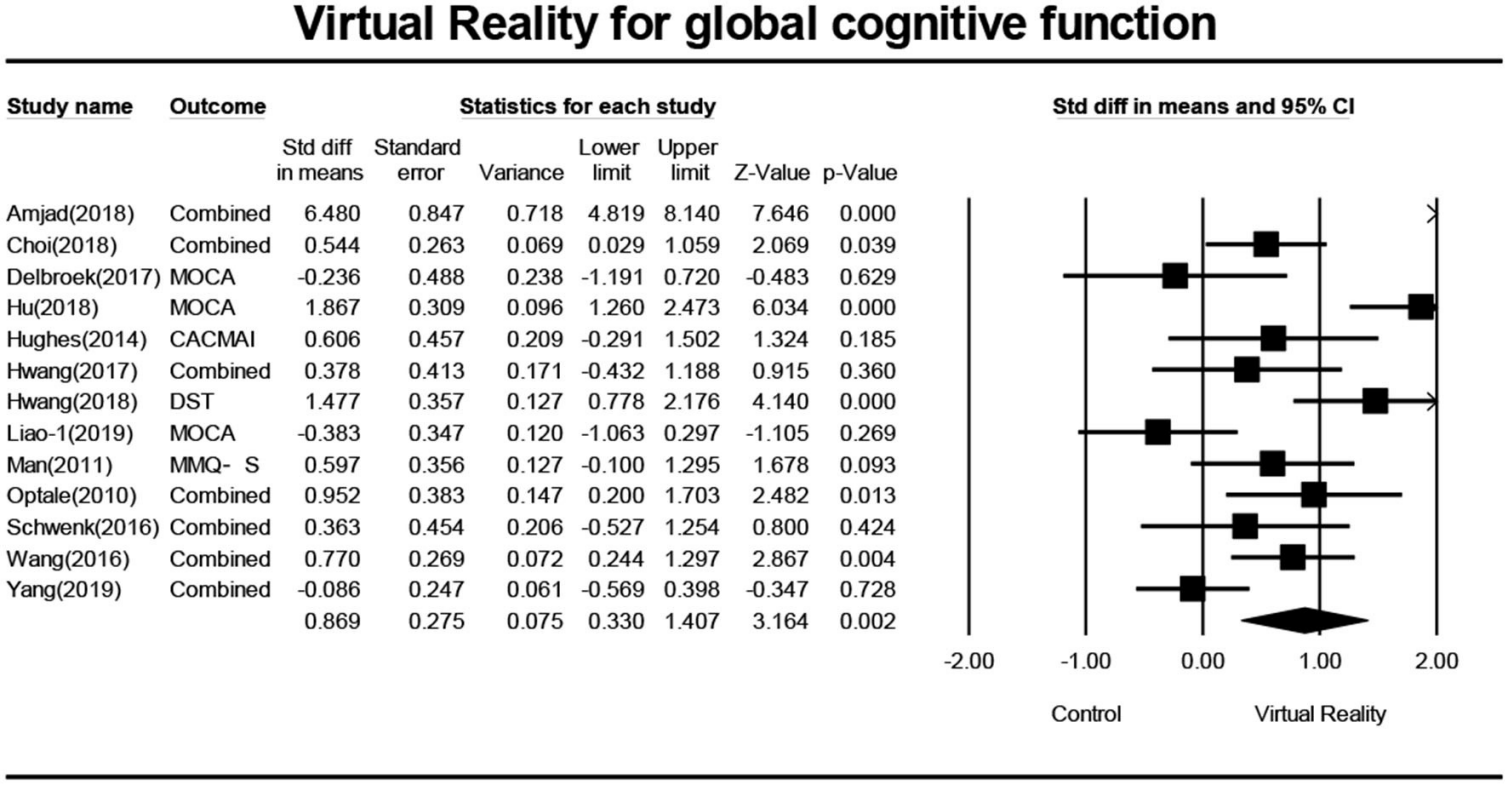
The effect of VR on the overall cognitive function of the MCI group (forest plot).

Thirteen studies (Optale et al., 2010; Man et al., 2012; Hughes et al., 2014; Schwenk et al., 2016; Wang and Lv, 2016; Delbroek et al., 2017; Hwang and Lee, 2017; Amjad et al., 2018; Hu et al., 2018; Hwang and Park, 2018; Choi and Lee, 2019; Liao Y. et al., 2019; Yang et al., 2019) investigated the effect of VR on the overall cognitive function of MCI patients, and a total of 16 data were obtained. Among those, each of the seven studies (Optale et al., 2010; Schwenk et al., 2016; Wang and Lv, 2016; Hwang and Lee, 2017; Amjad et al., 2018; Choi and Lee, 2019; Yang et al., 2019) includes two pieces of data. 10 studies (Man et al., 2012; Hughes et al., 2014; Schwenk et al., 2016; Wang and Lv, 2016; Delbroek et al., 2017; Hwang and Lee, 2017; Hu et al., 2018; Choi and Lee, 2019; Liao Y. et al., 2019; Yang et al., 2019) reported the mean and standard deviation of the VR group and control group before and after intervention. One study (Hwang and Lee, 2017) reported the changes in the mean and standard deviation of the intervened VR group and control group before and after the intervention. One study (Optale et al., 2010) reported the *F*-value and *P*-value of the VR group and control group before and after the intervention, and one study (Amjad et al., 2018) reported the mean and standard deviation of the VR group and control group after intervention. After merging the same studies, 13 pieces of data were obtained and were subject to meta-analysis. Next, the visual funnel plot was symmetrically presented (Egger regression intercept = 4.434, *P* = 0.123, Figure 2). Further, we performed a sensitivity analysis on these variables; the leave-one-out sensitivity analysis result demonstrated that no removal of any single study could lead to a substantial change in pooled results (SMD = 0.869, 95% CI = 0.330–1.407). Meta-analysis indicated that, after VR training, the overall cognitive function of MCI patients was significantly improved (SMD = 0.869, 95% CI = 0.330–1.407, *P* = 0.002, *I*^2^ = 86.822, Figure 3).

### Effects of VR on Executive Function

**Figure 4:**
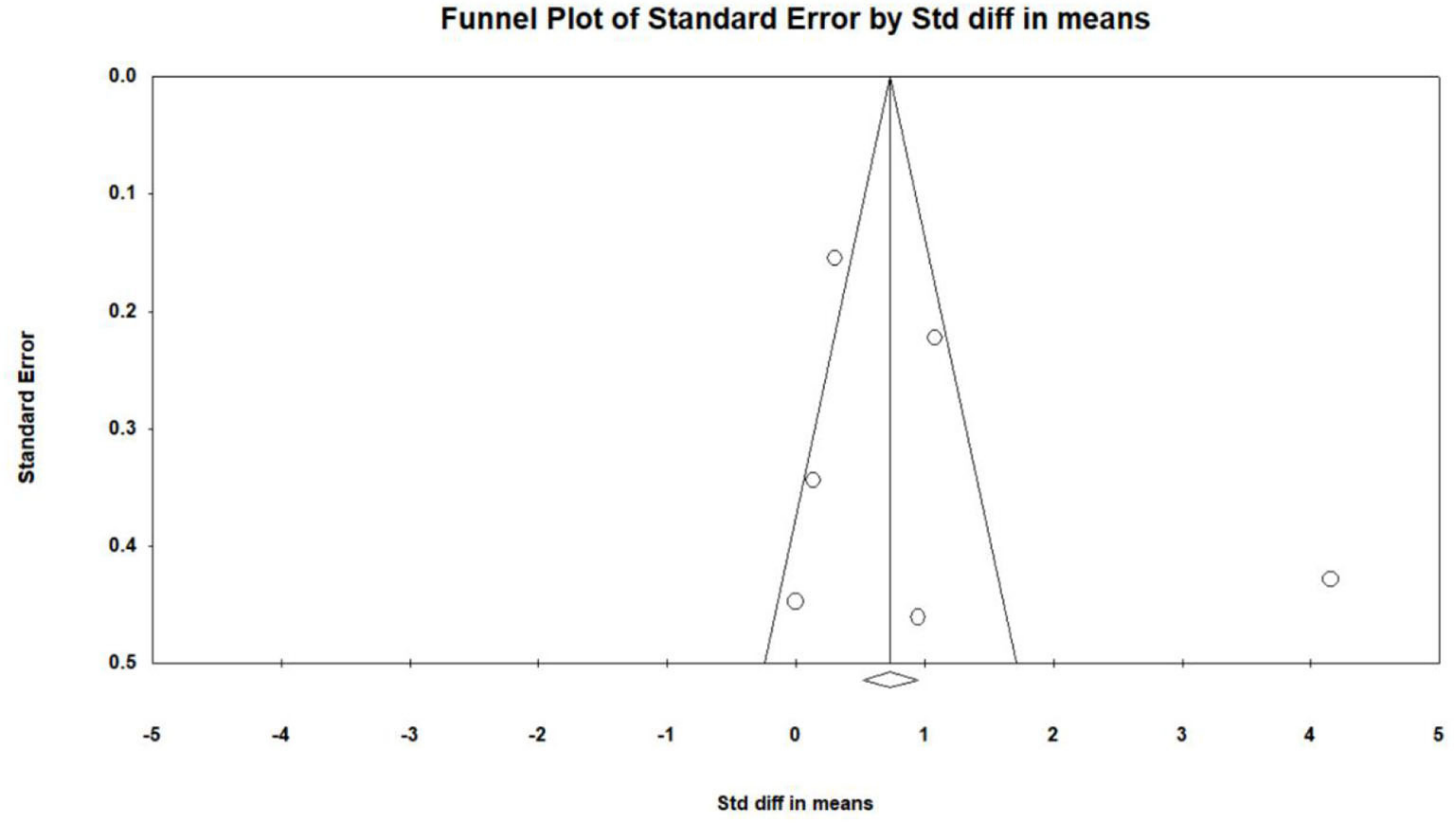
The publication bias of executive functions (funnel plot).

**Figure 5:**
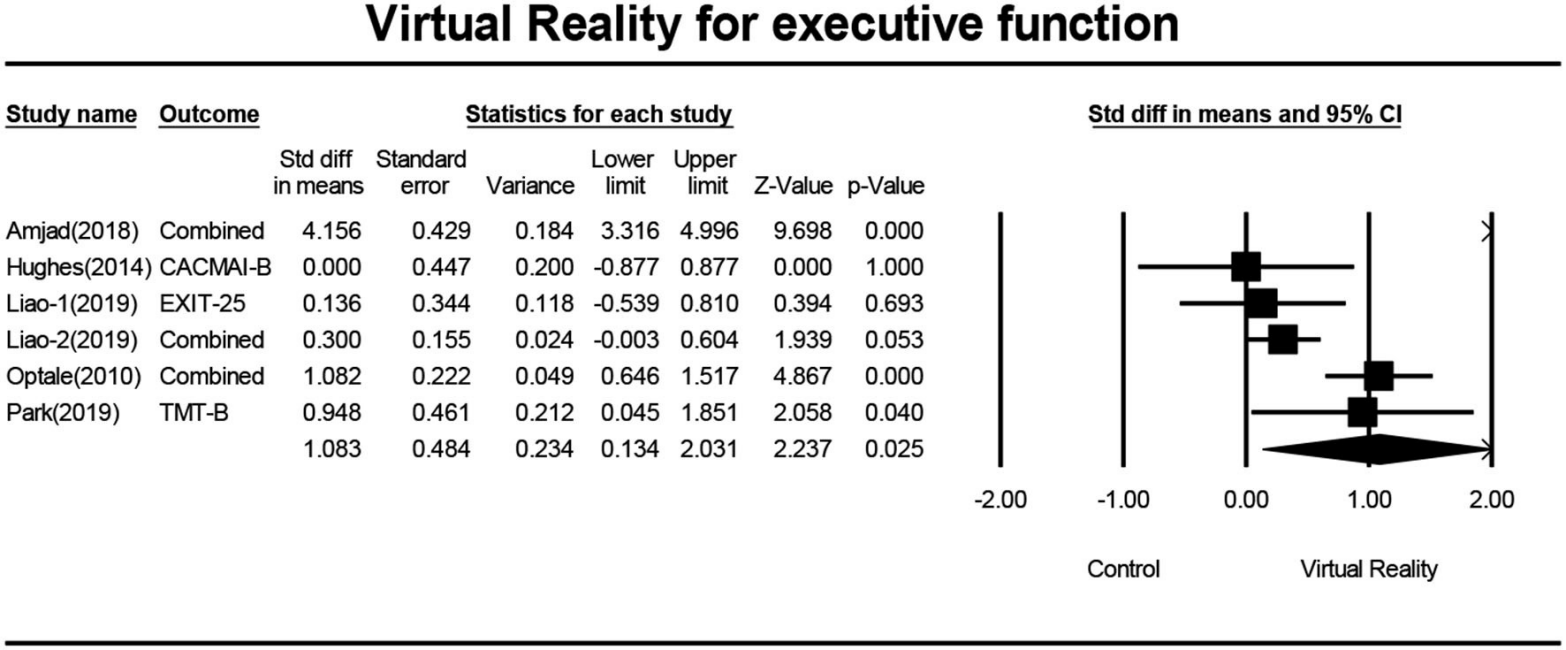
The effect of VR on executive functions of the MCI group (forest plot).

Six studies (Optale et al., 2010; Hughes et al., 2014; Amjad et al., 2018; Liao Y. et al., 2019; Liao Y. Y. et al., 2019; Park et al., 2019) investigated effects of VR on executive functions of MCI patients, and a total of 13 pieces of data were obtained. For these, each of the three studies (Optale et al., 2010; Amjad et al., 2018; Liao Y. et al., 2019) includes two pieces of data. Four studies (Hughes et al., 2014; Liao Y. et al., 2019; Liao Y. Y. et al., 2019; Park et al., 2019) reported the mean and standard deviation of the VR group and control group before and after the intervention. One study (Optale et al., 2010) reported the F value and P-value of the VR group and control group before and after the intervention. One study (Amjad et al., 2018) reported the mean and standard deviation of the VR group and control group after intervention. After merging the same studies, five pieces of data were obtained and were subject to meta-analysis. Next, the sensitivity analysis and funnel plots showed the existence of publication bias (Egger regression intercept = 3.723, *P* = 0.402, Figure 4). Further, we performed a sensitivity analysis on these variables; leave-one-out sensitivity analysis results demonstrated that no removal of any single study could lead to a substantial change in pooled results (SMD = 1.083, 95% CI = 0.134–2.031). Meta-analysis indicated that, after VR training, the executive functions of MCI patients were significantly improved (SMD =1.083, 95%CI = 00.134–2.031, *P* = 0.025, *I*^2^ = 93.748, Figure 5).

### Effects of VR on Short-Term Memory

**Figure 6:**
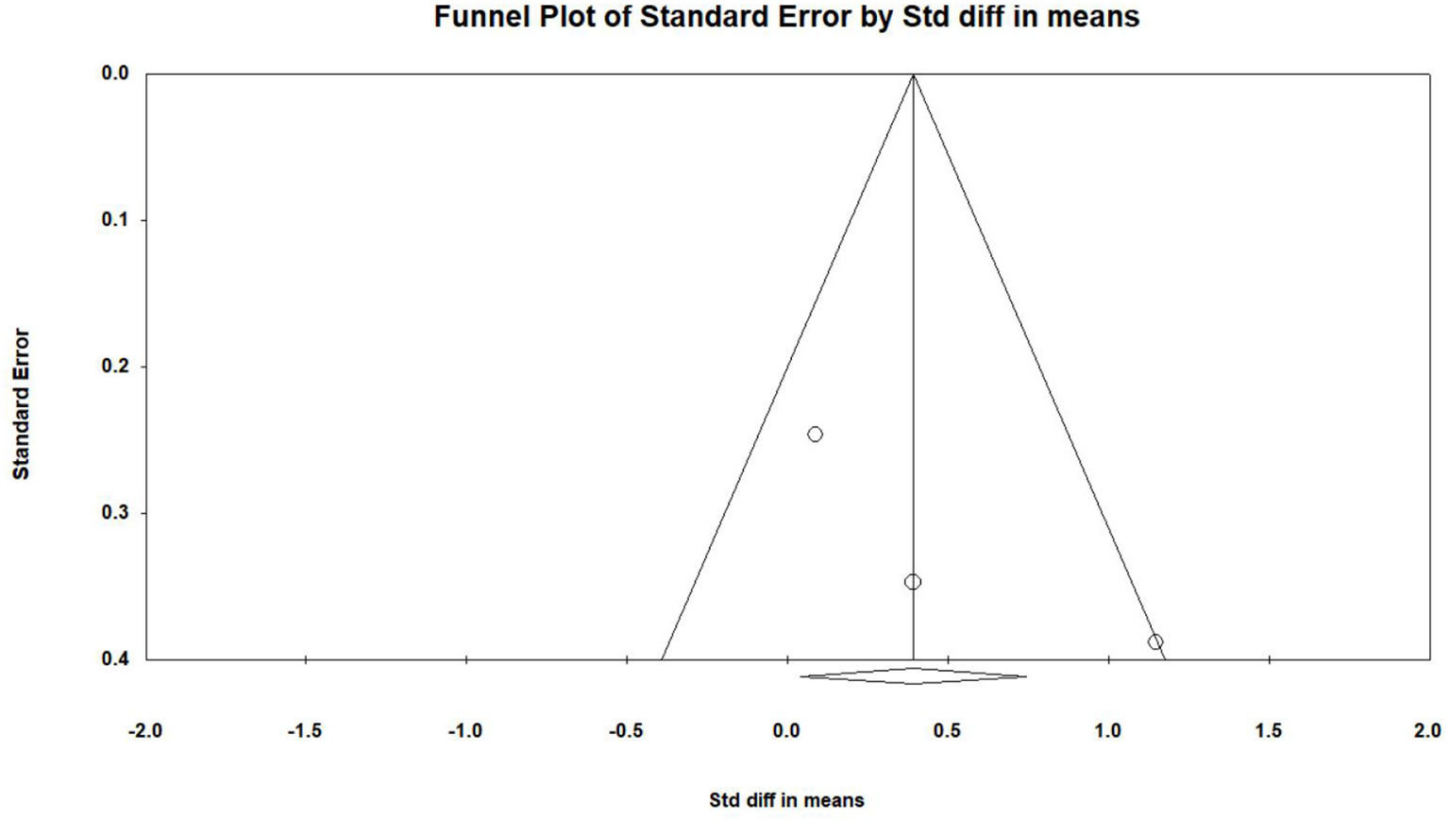
The publication bias of short-term memory (funnel plot).

**Figure 7:**
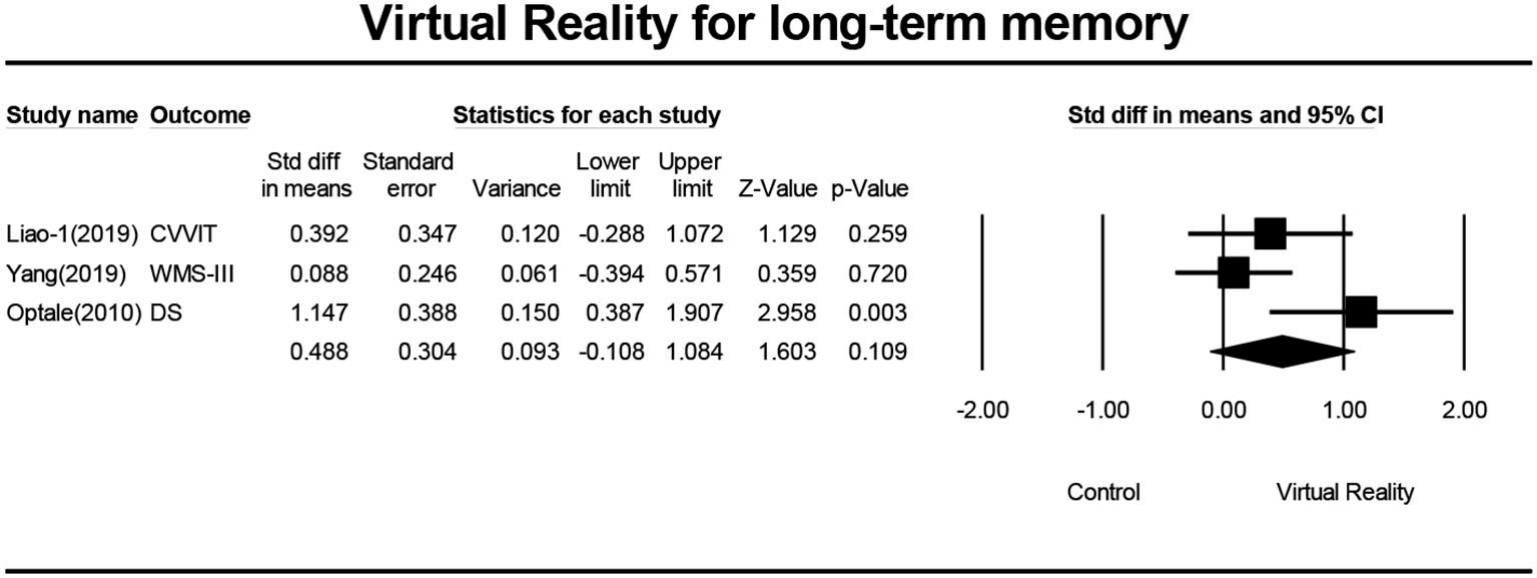
The effect of VR on short-term of the MCI group (forest plot).

Three studies (Optale et al., 2010; Liao Y. et al., 2019; Yang et al., 2019) investigated the effects of VR on the short-term memory of MCI patients, and a total of three pieces of data were obtained. Two studies (Liao Y. et al., 2019; Yang et al., 2019) reported the mean and standard deviation of the VR group and control group before and after the intervention. One study (Optale et al., 2010) reported the mean and standard deviation of the VR group and control group after intervention. Three pieces of data were subject to meta-analysis. Next, the sensitivity analysis and funnel plots showed publication bias (Egger regression intercept = 6.071, *P* = 0.286, Figure 6). Meta-analysis indicated that, after VR training, the short-term memory of MCI patients was not significantly improved (SMD = 0.488, 95%CI = −0.108–1.084, *P* = 0.109, *I*^2^ = 62.354, Figure 7).

### Effects of VR on Long-Term Memory

**Figure 8:**
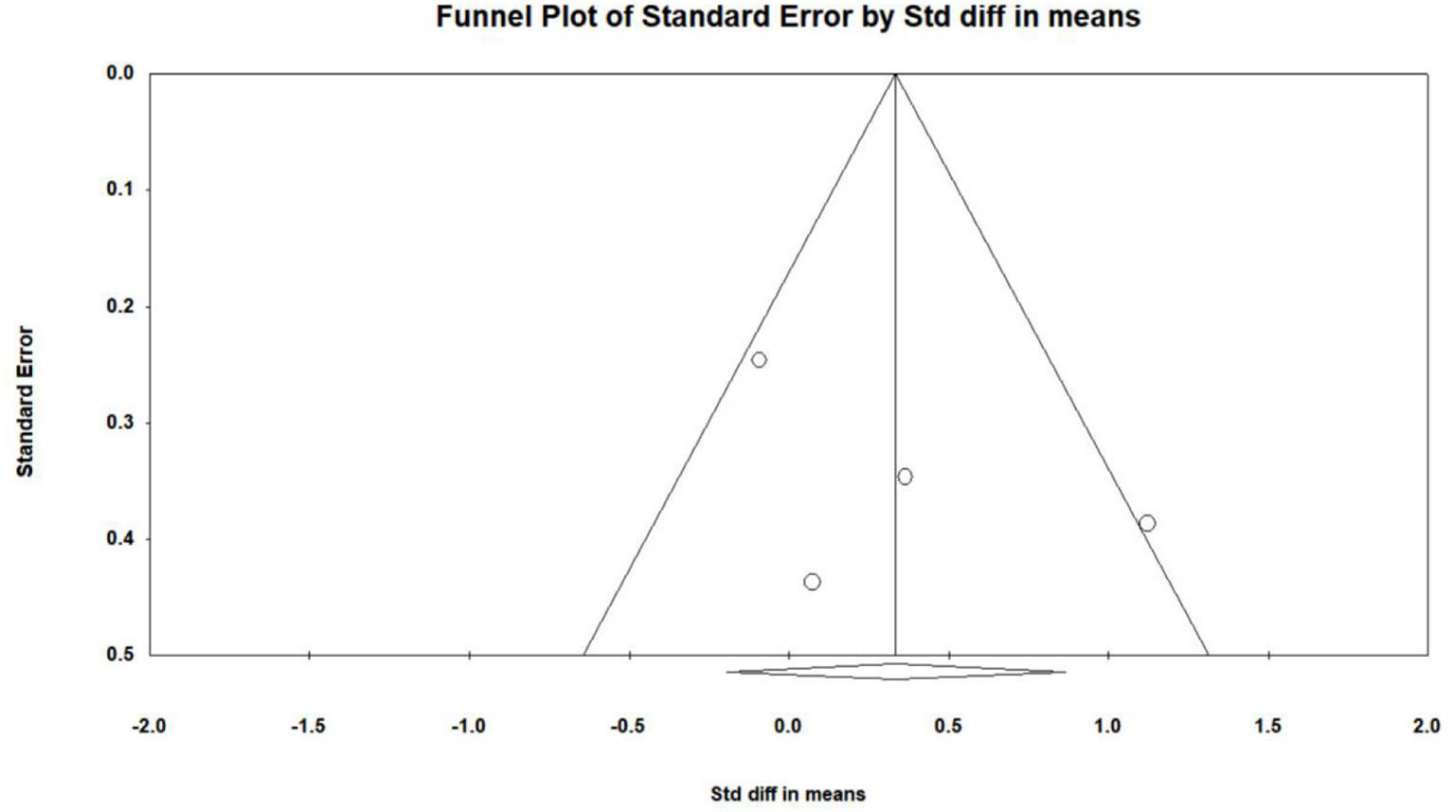
The publication bias of long-term memory (funnel plot).

**Figure 9:**
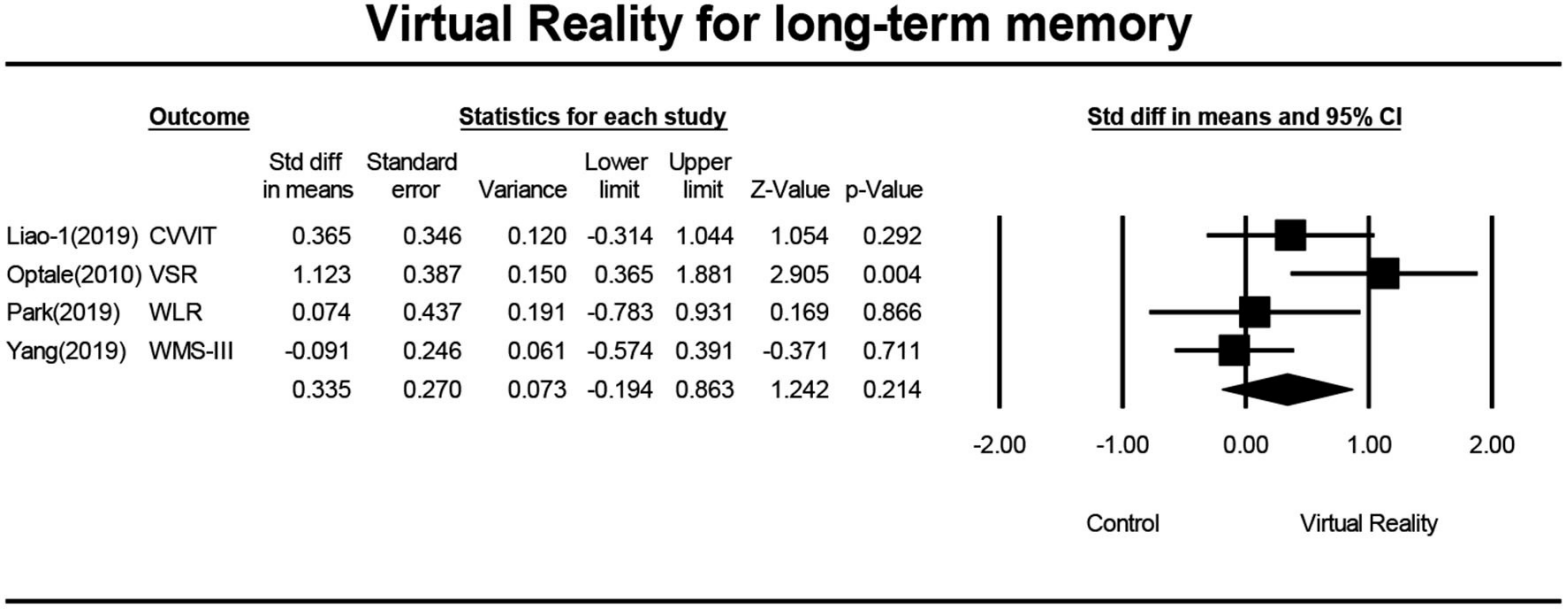
The effect of VR on long-term memory of the MCI group (forest plot).

Five studies (Optale et al., 2010; Liao Y. et al., 2019; Park et al., 2019; Yang et al., 2019) investigated the effects of VR on the long-term memory of MCI patients, and a total of five pieces of data were obtained. Four studies reported the mean and standard deviation of the VR group and the control group before and after the intervention. One study (Optale et al., 2010) reported the *F*-value and *P*-value of the VR group and control group before and after the intervention. Five pieces of data were first subjected to meta-analysis, and then to sensitivity analysis and funnel plotting. Statistics indicated that there was publication bias (Egger regression intercept = 7.50, *P* = 0.016, Figure 8). After removing the outlier (Man et al., 2012), a symmetrical funnel diagram was observed. Further, we performed a sensitivity analysis on these variables; leave-one-out sensitivity analysis result demonstrated that no removal of any single study could lead to a substantial change in pooled results (SMD = 0.335, 95% CI = −1.194–0.863). The meta-analysis indicated that, after VR training, the long-term memory of MCI patients was not significantly improved (SMD = 0.335, 95%CI = −1.194–0.863, *P* = 0.0.214, *I*^2^ = 58.868, Figure 9).

## Discussion

To the best of our knowledge, this study is the first meta-analysis on the effects of VR technology on the cognitive functions of MCI patients. Specifically, the present study shows that VR technology may help improve the cognitive functions of people suffering from MCI. Moreover, VR technology influences the cognitive functions of MCI patients to various degrees [overall cognitive function (SMD = 0.869) and executive functions (SMD = 1.083)]. It is worth noting that, although the effects on short-term memory and long-term memory were not statistically significant in some studies, the effect of VR improving MCI memory function also shows a positive trend.

VR technology has made possible the interaction between humans and VR, and combining physical training and cognitive training into VR seems to be an effective intervention method (Liao Y. Y. et al., 2019). The compensation model of brain plasticity shows that an aging brain can maintain its best cognitive functions by increasing the activation of the brain network (frontal, temporal, and parietal regions) (Lustig et al., 2009). The advantage of VR is that it provides timely feedback for MCI patients and increases the stimulation in their cognitive areas and motor areas, thereby improving cognitive functions and daily living skills (Coyle et al., 2015). In this meta-analysis, only the rehabilitative effects of VR on cognitive functions and executive functions were shown, while no effects could be shown for short-term and long-term memory. For short-term memory, this study only included three studies (Optale et al., 2010; Liao Y. et al., 2019; Yang et al., 2019) (three pieces of data) and only one (Liao Y. et al., 2019) of them showed a significant difference between VR and conventional treatment. No significant difference was shown in the other two studies (Optale et al., 2010; Yang et al., 2019). As a result, no statistical significance was observed. Specifically, one of the studies that did not show significant differences (Optale et al., 2010) adopted auditory therapy and encouraged MCI patients to sing and play musical instruments, which, to some extent, stimulated MCI patients' memory functions, resulting in similar rehabilitative effects for both groups. In the other study (Yang et al., 2019), for the control group, the intervention was reading e-books and playing games, and no significant difference was found in short-term memory either. Although no statistical significance was observed, compared with traditional treatments, VR treatment is more affordable, more personalized, and easy to operate. Only five studies (Optale et al., 2010; Liao Y. Y. et al., 2019; Park et al., 2019; Yang et al., 2019) on long-term memory were included in this review, and four (Optale et al., 2010; Liao Y. et al., 2019; Park et al., 2019; Yang et al., 2019) were kept for analysis. Three of them did not show statistical significance. Two of the studies investigated long-term memory and short-term memory and found no significant difference in both. This may be due to the active intervention (gaming, reading) used in the control group, which may have positive impacts on the improvement of memory (Liao Y. et al., 2019; Yang et al., 2019). As shown in the meta-analysis, compared to the control group, the long-term and short-term memory of the VR group were not improved.

### Global Cognitive Function

In this meta-analysis, the overall cognitive function was mainly determined by MMSE or MoCA, or the combined effect size. There were a few other adopted measuring tools (DST, MMQ, CACMAI). MMSE is the most widely used tool for assessing the overall cognitive function, while MoCA is a brief tool that can test the cognitive function more comprehensively and is more sensitive to testing MCI (Nasreddine et al., 2005). When examining an individual experiment, no significant difference was found in seven studies (Man et al., 2012; Hughes et al., 2014; Schwenk et al., 2016; Delbroek et al., 2017; Hwang and Lee, 2017; Liao Y. Y. et al., 2019; Yang et al., 2019), possibly owing to the VR group and the active control group. The improvement of global cognitive function in the VR group may be related to the reduction of brain activation in the prefrontal region (Liao Y. et al., 2019). It is noteworthy that researchers have found that, after receiving combined therapy (i.e., combined physical and cognitive interventions), the elderly with MCI were improved more prominently in various cognitive functions, compared with those only receiving cognitive or physical therapy (Barnes et al., 2013). The superiority of the application of VR technology comes from the integration of physical and cognitive training into VR. Some studies have suggested that dual cognitive and physical tasks based on VR technology preferably improve MCI cognition in both somatic function (such as muscle strength, balance, and endurance) and cognition function (such as attention, visual-spatial ability, and executive function) at a higher level than conventional rehabilitation training can provide (Liao Y. et al., 2019). Nevertheless, most studies related to VR training focus either on physical or cognitive training, and there is still a lack of research on the effects of combining these two kinds of training in the VR environment (Liao Y. et al., 2019). Thus, to research the effects of VR-based physical and cognitive training on the elderly with MCI, more evidence is needed. The overall meta-analysis result shows that intervention methods involving VR technology may have potential rehabilitative effects on the overall cognitive function of people with MCI.

### Executive Function

Executive functions are the advanced cognitive functions involved in planning, initiating, monitoring, and suppressing target-oriented behaviors, and are the basis for MCI patients' daily activities (Gilbert and Burgess, 2008; Zou et al., 2019). As for executive functions, the meta-analysis in this study shows that VR has a relatively positive impact on the executive functions of MCI patients, which may be related to the features of human-computer interaction in VR. The purpose of VR technology is to make the interaction between human and machine possible; while experiencing the simulated environment, the movement is sensed through sensor technologies like stereo display technology, and then the computer receives the feedback from the computer and data output, and in this way the human-machine interaction is completed (Tupa et al., 2015). As a result, in the virtual task, MCI's executive function seems to be improved by timely feedback to the switching between different tasks (Liao Y. Y. et al., 2019). When examining an individual experiment, no significant difference was found in three studies (Hughes et al., 2014; Liao Y. Y. et al., 2019), possibly because of the active intervention used in the control group, which may have positive impacts on the improvement of executive function. Due to the advantages of real-time feedback from VR, primarily for vision and attention, it can effectively promote MCI to perform complex executive functions. For example, in the kitchen virtual reality game, MCI needs to switch between different tasks to complete the task. Thus, various parts of MCI participants' executive function were trained, and the real-time feedback of MCI within VR may have a more significant impact on various executive functions (Liao Y. Y. et al., 2019). The overall meta-analysis result shows that intervention methods involving VR technology may have potential rehabilitative effects on the executive function of people with MCI.

### Practical Implications

The current meta-analysis suggests that VR technology may aid in slowing the progression of MCI to dementia by improving the overall cognitive functions and executive functions of the patient; this shows that VR intervention can play a positive role in various clinical results for patients with cognitive impairment, which means that the improvement of cognitive and routine function could be realized by VR intervention through the way of stimulating patients' brains. The findings in this study may provide evidence of VR's rehabilitative effects on MCI for clinicians. However, it is worth noting that in the studies included in this analysis, except for the one study (Choi and Lee, 2019) that involved specialized equipment (virtual rowing), all the studies used simple equipment (e.g., desktop, goggles-and-gloves, large screen, virtual room) to build the VR environment for MCI intervention, such as a virtual supermarket environment or virtual home environment. MCI patients were asked to participate in cognitive training (executive functions, attention, memory) (Huang et al., 2005; Man et al., 2012; Park et al., 2019). Through various tasks, specific brain regions were likely “activated,” and thus, their cognitive functions were improved. On the other hand, VR offers cost-effective, accessible, flexible, and comprehensive interventions for patients who have difficulty attending outpatient appointments due to distance, lack of transport, or disability. In addition, VR integrates real-time computer graphics, body tracking devices, visual displays, and other sensory inputs, which can be utilized to provide long-term and individualized care for patients with dementia. VR treatment has advantages compared with traditional treatment, such as it being more affordable and more personalized. The VR equipment is relatively expensive, but researchers are trying to use smartphones to create a virtual environment (Donker et al., 2019), which would mean future patients would only need to purchase VR glasses and can realize the treatment at home. The first attempt with VR devices may require the therapist's assistance, but in the long run, those within the patient's home could assist in operating the VR system, reducing the number of visits to the hospital, and becoming an easy-to-operate home treatment. However, due to the limited number of studies, no subgroup meta-analysis could be conducted. Therefore, further research on the training content, frequency, and intensity of VR treatment for MCI is needed.

### Study Limitations

The merit of this study is that it is the first meta-analysis on randomized controlled trials about the rehabilitative effects of VR technology on MCI patients. Moreover, by calculating the combined effect size, the influencing degree of VR technology on cognitive functions is confirmed. However, this study has some limitations. First, when the publication date was set on October 28th, 2019, research findings on this topic (ongoing research projects, manuscripts under review, and article published after the date) were possibly excluded from this study. Second, studies on the effects of VR technology on the cognitive behavior of MCI patients were only investigated, and related research on neural mechanisms was not explored. Third, this review included only 15 studies, which is a relatively small number. The main limitation of this study is that in the included studies, VR is not the only treatment, so it is difficult to say that all the improvements resulted from VR technology. Lastly, because the intervention plans included in the studies differ to a large extent, it is difficult to give suggestions on the specific duration and frequency of interventions. The above gaps should be resolved in future primary research.

## Conclusion

The current meta-analysis indicates that VR technology may have rehabilitative effects on the cognitive functions (overall cognitive function, executive function) of people with MCI and provide a better intervention method for MCI. Further studies need to be more in-depth and detailed so as to verify the benefits of VR technology on MCI patients' cognitive functions.

## Data Availability Statement

The datasets generated for this study can be found in the 1800371011@email.szu.edu.cn.

## Author Contributions

JW and ZR contributed to the conception, design of the review, applied the selection criteria, and completed assessment of risk of bias. JW applied the search strategy and wrote this manuscript. YM analyzed and interpreted the data and edited this manuscript. ZR was responsible for the overall project. All authors contributed to the article and approved the submitted version.

## Conflict of Interest

The authors declare that the research was conducted in the absence of any commercial or financial relationships that could be construed as a potential conflict of interest.
